# Understanding disparities in simulation through minority stress theory: a conceptual framework

**DOI:** 10.1186/s41077-026-00424-z

**Published:** 2026-02-24

**Authors:** Remy Roe

**Affiliations:** Department of Graduate Medical Education, HCA Healthcare, HCA Florida St. Lucie Hospital, Port St. Lucie, FL 34952 USA

**Keywords:** Minority stress, Equity, Diversity, Inclusion, Healthcare simulation

## Abstract

Despite growing attention to equity in healthcare simulation, persistent disparities in participation and learning outcomes among learners from minoritized backgrounds remain insufficiently explained. Existing simulation-based education frameworks describe how learning occurs but offer limited insight into how inequitable learning conditions shape learners’ experiences. We propose minority stress theory (MST) as a conceptual framework for understanding how stress related to minoritized identity operates within healthcare simulation environments. Originally developed to explain health disparities among sexual minorities, MST conceptualizes stress as arising from both structurally embedded conditions (distal stressors) and internalized vigilance and self-monitoring (proximal stressors). Through conceptual analysis, we map MST constructs onto core features of healthcare simulation, including its performance-based, socially intensive, evaluative, and immersive characteristics. We illustrate how minority stress processes may operate through education-relevant mechanisms, such as cognitive load, psychological safety, engagement, and help-seeking, to shape learning experiences and performance. By applying MST to simulation-based education, this paper offers an equity-informed framework for examining learning-environment mechanisms and informing inclusive simulation design, facilitation, and assessment practices.

## Why existing simulation frameworks fall short for equity

Healthcare simulation has emerged as a cornerstone of health profession education, with well-documented benefits [1–3]. However, persistent disparities in participation, engagement, and outcomes among learners from minoritized backgrounds remain inadequately understood [4, 5]. Learners from racial and ethnic minority groups, LGBTQ+ communities, lower socioeconomic backgrounds, and other marginalized identities often report feeling less included in simulation activities, experiencing greater anxiety, and demonstrating reduced engagement than their majority-group peers do [6–8].

Current approaches to equity in simulation (e.g., demographic representation in scenarios, cultural competence training, and general inclusive education principles) lack a cohesive theoretical framework to explain the psychological mechanisms by which minoritization affects simulation learning [9, 10]. Existing simulation frameworks (e.g., experiential learning theory, situated cognition) do not account for the unique stressors faced by minoritized learners [11–13]. Although they are generally diverse frameworks that are essential for values and awareness, they lack specificity for understanding the particular challenges of simulations.

Despite these efforts, what remains undertheorized is how stress related to minoritization is produced and experienced within simulation itself. Simulation-based education differs from many other learning environments in that performance is public, socially embedded, emotionally engaging, and often evaluative. These features create conditions under which stress is not incidental but structurally embedded in the learning activity. Existing simulation learning theories describe how learning occurs but offer limited insight into how inequitable stress processes may be differentially activated for minoritized learners within these environments.

The unique characteristics of healthcare simulations may particularly amplify minority stress in ways that distinguish it from other educational contexts. Unlike didactic learning, where performance remains largely private, simulation makes learners’ clinical reasoning, communication, and technical skills immediately visible to peers and faculty, creating heightened vulnerability for those already concerned about stereotype confirmation. Moreover, the fidelity and emotional engagement of simulation demand real-time identity negotiation that written assessments or low-stakes discussions do not.

We therefore propose minority stress theory (MST) as a theoretical lens well suited to explaining how structurally embedded features of simulation-based education may differentially produce stress for minoritized learners. MST adds an explicitly equity-informed psychosocial perspective to simulation scholarship by elucidating how stress processes related to minoritized identity interact with simulation’s experiential, social, and evaluative dimensions. While experiential learning theory and situated cognition provide valuable foundations for understanding how learners construct knowledge through simulation, they primarily describe general learning processes rather than the inequitable conditions under which learning occurs. In this way, MST does not replace existing simulation theories but rather enriches them by revealing mechanisms through which learning environments may differentially affect learners.

Initially developed by Meyer to explain mental health disparities among sexual minorities [14, 15], MST posits that individuals from stigmatized groups experience chronic stress arising from both objective discrimination (distal stressors) and internalized expectations and vigilance (proximal stressors). This stress is additive to general life stressors and affects psychological well-being, cognitive functioning, and social engagement [16]. While MST necessarily focuses on stress processes and barriers, this lens should not obscure the considerable strengths minoritized learners bring that enrich learning environments when those environments are structured to support rather than burden them.

The performance-based nature of simulation can trigger stereotype threat [17, 18]. Social intensity heightens concerns about belonging [19]. Evaluative components activate anxieties about biased assessment [20]. Immersive quality forces minoritized learners to confront tensions between personal identities and professional roles. While general diversity frameworks in medical education address representational equity and cultural competence, they have not adequately theorized the psychological mechanisms through which simulation’s specific characteristics (e.g., observable performance, social intensity, evaluative stakes, and immersive realism) may disproportionately burden minoritized learners. Understanding these dynamics through MST provides simulation educators with a nuanced approach to creating equitable learning environments.

This paper maps MST onto healthcare simulation through detailed conceptual analysis, identifying both distal stressors (objective discrimination) and proximal stressors (internalized expectations) affecting minority learners. We examine the pathways through which these stressors affect learning, identify resilience factors, outline research directions, and acknowledge limitations. Our intention is not to supplant other equity frameworks but rather to offer a complementary perspective that deepens understanding of minoritized learners’ psychological experiences.

By revealing the additional cognitive and emotional burdens that minority stress places on learners, we hope to inspire theoretically grounded approaches to inclusive simulation design. The timing is critical; as healthcare systems grapple with health inequities and educational institutions diversify, simulation educators have both the opportunity and the responsibility to ensure simulation advances rather than reproduces patterns of exclusion. This paper focuses on learning-environment mechanisms rather than proposing comprehensive structural reform.

## Minority stress theory: core concepts

In this section, we outline core elements of minority stress theory (MST) that are directly relevant to understanding learning experiences in simulation-based education, rather than providing a comprehensive review of the theory. MST was developed to explain persistent mental health disparities among sexual minorities that cannot be accounted for by individual pathology alone [21]. Meyer’s formalization of the theory linked subordinate social positioning within social hierarchies to health disparities through specific, socially produced stress processes [14, 15]. The central premise of MST is that individuals occupying minoritized positions experience unique stressors, or minority stress, that are additive to general life stressors and that accumulate over time, undermining well-being [15].

MST builds on transactional models of stress [22] by emphasizing that certain stressors are uniquely tied to one’s social position within hierarchical systems. These stressors are chronic rather than episodic, socially produced rather than individually generated, and structurally patterned rather than random [14]. A key contribution of MST is Meyer’s distinction between *distal* and *proximal* minority stressors, which differentiates between objective, external stressors embedded in the environment and subjective, internal processes shaped by sustained exposure to stigma [14, 15].

### Distal and proximal stressors in simulation

Distal minority stressors refer to objective, external events and conditions that occur by virtue of group membership, independent of individual perception. In the context of sexual minorities, these include discrimination, exclusion, harassment, violence or threat of violence, and structural stigma reflected in policies and institutional practices [15, 23]. Because distal stressors are embedded in the environment, they cannot be addressed solely through individual coping or cognitive reframing but require attention to structural and contextual conditions [15]. Within simulation-based education, distal stressors may manifest through differential evaluation, biased role assignment, inequitable feedback practices, or institutional norms that signal whose performance is expected or valued.

Proximal minority stressors involve subjective processes related to identity, self-perception, and appraisal. Meyer identifies three primary proximal stress processes: expectations of rejection, concealment, and internalized stigma [14, 15]. Expectations of rejection involve chronic vigilance for potential bias, requiring sustained cognitive and emotional resources. Concealment reflects the ongoing effort to monitor behavior, language, or disclosure of identity in order to avoid stigma. Internalized stigma refers to the incorporation of negative societal attitudes into one’s self-concept, which can undermine confidence and self-efficacy [14]. In simulation settings, where performance is visible, time-pressured, and socially evaluated, these proximal stressors may be particularly activated, diverting cognitive resources away from learning and task execution toward impression management and self-monitoring.

MST further specifies pathways through which distal and proximal stressors affect outcomes, including depletion of psychological resources and disruptions in cognitive, emotional, and social functioning [15]. Mediating processes such as appraisal, coping, and access to social support influence how stressors are experienced and managed [15, 22]. Importantly, MST also identifies resilience-related processes, including community connectedness, positive identity, and collective resistance to stigma [15, 24]. In educational contexts, these processes may be shaped by learning climate, peer interactions, and instructional design choices that either buffer or exacerbate stress exposure.

Although originally developed in relation to sexual minorities, MST has been extended to other minoritized groups, including racial and ethnic minorities, transgender individuals, people with disabilities, immigrants, and religious minorities [25–29]. These applications retain the theory’s core structure while attending to context-specific stressor forms. Healthcare education represents a particularly salient context for minority stress due to its high-stakes nature, emphasis on professional identity formation, and historical patterns of exclusion [30, 31]. Simulation-based education may further intensify these dynamics, as simulations are performance-based, socially intensive, evaluative, and emotionally engaging [32]. MST offers a framework for understanding how learners enter simulation with cumulative exposure to stigma and how simulation activities may differentially activate distal and proximal stress processes, shaping learning experiences and outcomes.

## Minority stressors in healthcare simulation

**Fig. 1:**
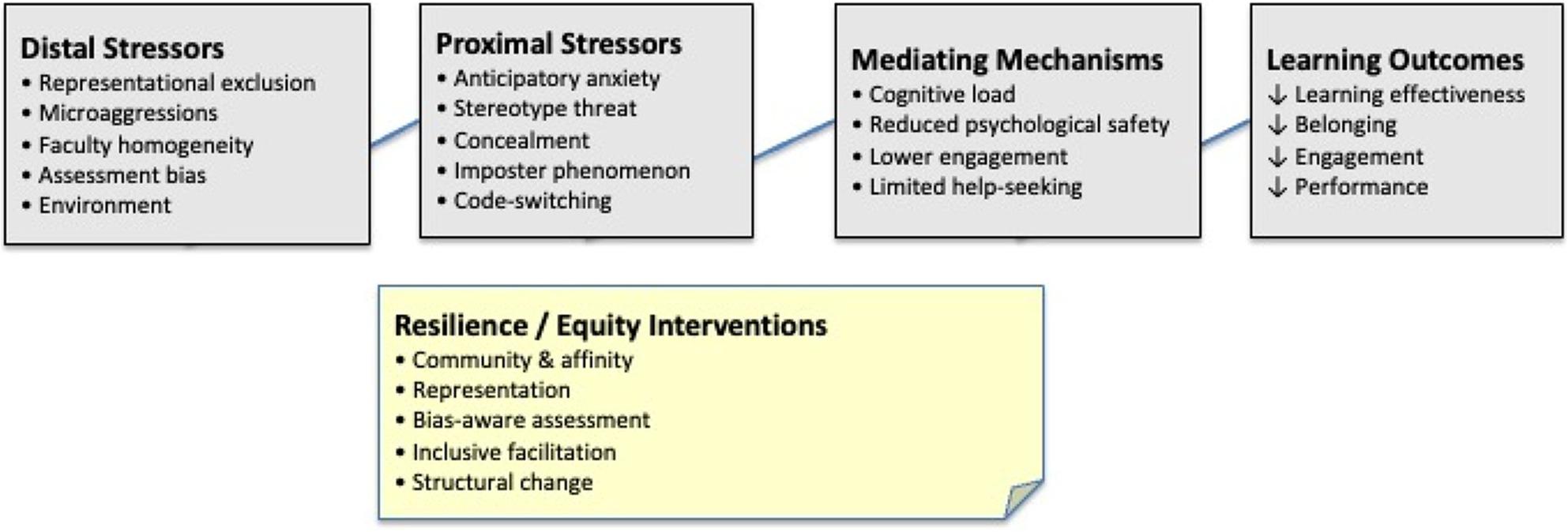
Conceptual model of minority stress processes in healthcare simulation. Distal and proximal stressors influence learning through mediating mechanisms such as psychological safety and engagement. Resilience and equity interventions mitigate these effects, promoting equitable learning outcomes

We now map minority stress constructs onto healthcare simulation-specific features, illustrating how stressors are produced, experienced, and mediated within simulation-based learning environments. Figure 1 provides an overview of how structurally embedded stressors in simulation give rise to psychosocial stress processes that operate through key mediating mechanisms to shape learning experiences and outcomes.

The figure illustrates how structurally embedded features of simulation-based education (distal stressors; left) give rise to psychosocial stress processes (proximal stressors; center) among minoritized learners. These stress processes operate through key mediating mechanisms (e.g., cognitive load, psychological safety, engagement, and help-seeking) that shape learning experiences and performance. Consistent with minority stress theory, stressors are additive to general demands and reflect both environmental conditions and internalized vigilance. Resilience-related factors and equity-informed design choices (bottom) may buffer stress effects by altering environmental conditions and supporting adaptive coping, thereby mitigating downstream impacts on learning outcomes.

### Structurally embedded stressors in simulation

As shown on the left side of Fig. 1, simulation environments may produce distal stressors through representational, interpersonal, and institutional features.

#### Representational exclusion

When scenarios consistently feature majority-group patients, minoritized learners receive implicit messages about whose health concerns are centered on [33]. In addition to their absence, minoritized groups often appear stereotypically. For example, patients of color are disproportionately depicted as being involved in substance use or noncompliance; LGBTQ+ patients are only involved in sexual health scenarios; and lower socioeconomic patients are portrayed as neglectful [34, 35]. These patterns create stress by seeing communities stereotyped, experiencing a lack of positive representations, and witnessing peers’ reactions that reveal biases.

#### Microaggressions

Microaggressions [36] are prevalent in simulations because of their interactive, unscripted nature. In these scenarios, learners may make biased assumptions about standardized patients based on race, accent, or appearance. Debriefings can become sites of microaggressions through comments such as “I did not even notice their race” (denying significance), “You’re so articulate” (expressing surprise at competence), or “Where are you truly from?” (implying foreignness) [37]. Microaggressions accumulate to create chronic stress with measurable psychological and physiological responses [38].

#### Faculty homogeneity

Faculty homogeneity constitutes distal stress when minoritized groups are underrepresented in leadership and facilitation [39]. This limits access to role models and mentors, communicates who is authorized to teach, and reduces the likelihood of recognizing and interrupting bias during activities [40].

#### Assessment bias

Assessment bias represents a particularly consequential form of distal stress. Minoritized learners are often evaluated more harshly for equivalent performance [41]. In simulation, this manifests as more intense questioning of clinical decisions, evaluation of different communication styles as less professional, and subjective criteria such as “leadership presence” that provide opportunities for implicit bias [20].

#### Physical environments

Physical environments communicate through visual representations [42]. Suppose displayed photographs feature exclusively majority-group professionals, equipment includes only light-skinned manikins, and spaces lack universal accessibility. These are all signals that certain bodies fully belong, while others do not.

### Psychosocial stress processes activated during simulation

These structurally embedded stressors activate proximal stress processes during simulation, illustrated in the center of Fig. 1.

#### Anticipatory anxiety and hypervigilance

Minoritized learners may enter a simulation with heightened anxiety about potential discrimination [15]. This learned response creates chronic vigilance, or the constant monitoring of environments for threats, assessing safety, and preparing defenses. Hypervigilance depletes cognitive resources that would otherwise be devoted to learning [43]. Simultaneously engaging in clinical content while monitoring social threats increases cognitive load, reducing working memory for clinical reasoning and skill performance [44]. Even in objectively supportive environments, vigilance persists because power differentials make relaxing guards feel dangerous.

#### Stereotype threat

When minoritized learners are aware of negative stereotypes about their group’s abilities (stereotypes about women’s leadership, assumptions about intellectual abilities based on race, doubts about disabled individuals’ clinical competence), this awareness can undermine performance even when they reject stereotypes personally [17, 18]. Stereotype threat operates through cognitive interference. Concern about confirming stereotypes creates intrusive thoughts that disrupt attention [45]. A simulation’s emphasis on observable, real-time performance in front of others amplifies stereotype threat by making actions visible and increasing group identity salience [17, 18].

#### Concealment and disclosure

For minoritized learners whose identities are not immediately visible (LGBTQ+ learners, learners with nonapparent disabilities, and learners from lower socioeconomic backgrounds), simulation contexts create ongoing disclosure decisions. Decision-making itself constitutes cognitive and emotional labor, assessing risks and benefits and monitoring behavior to avoid inadvertent disclosure [46]. Concealment involves the following psychological costs: self-monitoring, fear of discovery, and a sense of inauthenticity [47]. Conversely, disclosure creates vulnerability to discrimination or stereotyping.

Imposter 

#### Imposter phenomenon

Many minoritized learners experience persistent self-doubt about their abilities despite evidence of objective competence [48]. While not unique to minoritized groups, it is significantly more prevalent among underrepresented individuals, particularly as tokens [49]. In simulation, the imposter phenomenon manifests as disproportionate anxiety about mistakes, attributing success to luck rather than competence, excessive self-criticism, reluctance to take leadership roles, and hesitance to voice opinions [50]. Each simulation becomes a test of whether one truly belongs, with mistakes confirming feared fraudulence.

#### Code-Switching

Minoritized learners whose natural communication styles differ from majority-group professional norms may feel pressure to modify self-presentation by code-switching [51]. Code-switching serves protective functions but creates cognitive load by requiring conscious, real-time monitoring and behavioral adjustment. In complex scenarios that already demand cognitive resources, the additional burden of code-switching can impair clinical performance and create psychological costs related to authenticity [43].

#### Cognitive load

Cognitive load represents a primary mediating mechanism [44]. Both distal and proximal stressors consume cognitive resources, reducing working memory capacity for clinical reasoning, skill execution, and learning [43]. When minoritized learners simultaneously manage clinical scenarios and navigate minority stress, they operate under divided attention that majority-group peers do not face. Importantly, cognitive depletion may make minoritized learners appear less competent than they are, and assessment may conflate minority stress effects with clinical knowledge.

#### Psychological safety

When minoritized learners experience or anticipate discrimination, their perception of psychological safety (the belief that one can take interpersonal risks without negative consequences) diminishes [32, 52]. This perception leads to behavioral adaptations that protect against social risk but limit learning. For example, minoritized learners may volunteer less often for leadership roles, hesitate to voice disagreement, and are reluctant to acknowledge uncertainty.

#### Engagement and immersion

Minority stress interferes with psychological immersion by pulling attention from clinical content toward social and identity concerns. A learner preoccupied with monitoring for microaggressions or managing stereotype threat cannot be fully present. Reduced engagement creates inequity, and minoritized learners effectively receive less educational benefit from the same experiences [53].

#### Help-Seeking

When concerned with confirming stereotypes or being perceived as less capable, admitting uncertainty becomes socially risky. Minoritized learners may hesitate to ask for clarification, avoid seeking assistance, or be reluctant to ask follow-up questions, limiting learning opportunities [54].

MST emphasizes protective factors essential for designing experiences that foster belonging [15]. Community and affinity groups connecting minority learners reduce isolation [23]. Representation in faculty and scenarios provides counternarratives to stereotypes and communicates a sense of belonging [39, 42]. Transparent assessment processes with structured rubrics and clear criteria mitigate anxiety about bias. Explicit inclusion commitments acknowledging minority stress realities validate minority learners’ experiences and reduce isolation. Distal and proximal stressors influence learning through mediating mechanisms such as psychological safety and engagement. Resilience and equity interventions mitigate these effects, promoting equitable learning outcomes.

## Implications for practice and future work

This conceptual analysis reveals that minority stress operates through multiple interconnected pathways in simulations. The framework has specific implications across multiple domains that require systematic implementation. We briefly outline key implications to guide future implementation efforts. For example, scenario design should deliberately diversify patient representations across clinical presentations, avoid stereotypical portrayals, and strategically address bias as learning content when carefully facilitated [5, 9]. Facilitation must intentionally foster psychological safety [32], honor multiple perspectives without positioning minoritized learners as group representatives, skillfully navigate difficult moments, and operate from a place of cultural humility [55].

Assessment should use structured criteria examined for cultural specificity, employ multiple assessors with bias calibration [56], emphasize formative feedback, and incorporate self-assessment. Environments require auditing physical spaces for inclusivity signals [42], investing in diverse equipment, ensuring universal accessibility [57], and establishing clear policies with accessible reporting mechanisms. Faculty development must build equity-informed facilitation competencies and prioritize diverse types of faculty recruitment and retention [39].

Finally, program-level commitments demand explicit equity statements with accountability, meaningful incorporation of learner voice, and coalition building across organizational boundaries. Coherence across domains stems from MST’s focus on understanding and addressing unique psychological burdens minoritization creates, providing a clear rationale for why particular practices matter.

## Research directions

This framework’s validity and utility must be empirically tested. Descriptive research should establish the prevalence and forms of minority stressors through surveys, interviews, and focus groups with minoritized learners; scenario content analyses should document representational patterns; and faculty perspective research should assess equity knowledge and practices. Correlational research should test whether minority stress predicts performance and learning through proposed pathways (cognitive load, psychological safety, engagement, and help-seeking) and examine whether stressors operate similarly across different minority identities, while accounting for intersectionality.

Intervention research should evaluate whether equity-informed practices reduce minority stress and improve outcomes; test scenario design interventions; facilitate training; assess innovations; and develop comprehensive program-level initiatives.

Measurement development is foundational, adapting and validating minority stress scales for simulation, establishing psychometric properties of outcome measures, and developing valid psychological safety and climate measures.

Throughout, community-engaged approaches should involve minorized learners as partners in all research phases, recognizing that those experiencing inequity possess invaluable expertise. Importantly, programs need not wait for definitive evidence before implementing equity-informed practices grounded in decades of minority stress research in other contexts [14, 15].

## Limitations

No single framework captures the full complexity of equity issues. Minority Stress Theory’s organizing logic centers on stress and pathology, risking deficit narratives about minoritized communities that bring valuable assets, such as lived healthcare system experiences, cultural knowledge, and heightened awareness of social dynamics. Future work should integrate asset-based frameworks, such as community cultural wealth [58], to achieve a more balanced understanding.

While MST acknowledges that stress arises from social structures, its primary unit of analysis is individual psychological experience, thereby locating problems and solutions at the individual level. If programs respond by offering resilience training to minority learners while leaving inequitable structures intact, the framework may be misapplied. Future work must maintain a clear focus on structural and systemic changes as primary intervention targets [59].

MST was initially developed for single-axis minority status and may not adequately capture the unique experiences of individuals with multiple minority statuses. A black woman does not experience racism and sexism as separate additives but rather the unique oppression of gendered racism [60]. Future development must more robustly incorporate intersectionality. MST was developed in Western individualistic cultural contexts. International application requires careful consideration of cultural appropriateness and adaptation, involving scholars from diverse global contexts.

Translating the framework into measurable constructs presents challenges. Minority stress involves subjective experiences that are difficult to quantify, and measuring it could itself become a source of stress. Applying MST requires identifying who counts as minorized, risks essentializing identity categories. There is a risk that frameworks could be misappropriated for surveillance rather than liberation.

Minority Stress Theory should not supplant other important perspectives but should serve as one lens among several. Integration with critical race theory [61], feminist and queer theory [62], disability studies [63], and cultural wealth frameworks creates a more comprehensive understanding. The framework is most valuable when it is integrated with complementary perspectives, applied with cultural awareness, and implemented with a genuine commitment to structural transformation.

## Conclusion

This paper has introduced minority stress theory to the simulation community as a framework for understanding the barriers to participation faced by minority learners. Through conceptual analysis, we mapped minority stress constructs onto simulation-specific features, identifying how their unique characteristics amplify both distal stressors (objective discrimination) and proximal stressors (internalized expectations and vigilance).

MST offers the simulation community nuanced language to describe the multilayered psychological burdens that minority learners carry, identifies concrete mechanisms through which simulation may activate minority stress, and positions equity as central to educational quality. Minority stress operates through mechanisms that directly impair learning. The framework challenges examination of taken-for-granted practices and underscores that creating equitable simulation requires specific knowledge, concrete skills, and sustained organizational commitment beyond good intentions.

We envision a simulation where minoritized learners experience genuine belonging, where their participation is not burdened by additional stress, where scenarios reflect community diversity, where facilitation honors all voices, where assessment is transparently fair, and where environments communicate unambiguous inclusion. Achieving this requires commitment to ongoing learning and humility, centering minoritized voices, structural change over individual remediation, accountability and transparency, and sustained effort over performative gestures.

The work of creating equitable simulations is urgent, ongoing, and collective. It requires honesty about current practices, courage to change systems, and commitment to centering those marginalized. Minority stress theory provides a valuable framework that can catalyze conversation, critique, and action toward transforming simulation into a space where all learners belong and can contribute their full talents.

## Data Availability

No datasets were generated or analysed during the current study.
